# GOLPH2 protein expression as a novel tissue biomarker for prostate cancer: implications for tissue-based diagnostics

**DOI:** 10.1038/sj.bjc.6604614

**Published:** 2008-09-09

**Authors:** G Kristiansen, F R Fritzsche, K Wassermann, C Jäger, A Tölle, M Lein, C Stephan, K Jung, C Pilarsky, M Dietel, H Moch

**Affiliations:** 1 Department of Surgical Pathology, University Hospital Zurich, Zurich, Switzerland; 2 Institute of Pathology, Charité - Universitätsmedizin Berlin, Berlin, Germany; 3 Department of Urology, Charité - Universitätsmedizin Berlin, Berlin, Germany; 4 Department of Surgery, University Hospital Dresden, Dresden, Germany

**Keywords:** GOLPH2, GP73, prostate cancer, diagnostic marker, immunohistochemistry

## Abstract

*GOLPH2* is coding the 73-kDa type II Golgi membrane antigen GOLPH2/GP73. Upregulation of *GOLPH2* mRNA has been recently reported in expression array analyses of prostate cancer. As GOLPH2 protein expression in prostate tissues is currently unknown, this study aimed at a comprehensive analysis of GOLPH2 protein in benign and malignant prostate lesions. Immunohistochemically detected GOLPH2 protein expression was compared with the basal cell marker p63 and the prostate cancer marker *α*-methylacyl-CoA racemase (AMACR) in 614 radical prostatectomy specimens. GOLPH2 exhibited a perinuclear Golgi-type staining pattern and was preferentially seen in prostatic gland epithelia. Using a semiquantitative staining intensity score, GOLPH2 expression was significantly higher in prostate cancer glands compared with normal glands (*P*<0.001). GOLPH2 protein was upregulated in 567 of 614 tumours (92.3%) and AMACR in 583 of 614 tumours (95%) (correlation coefficient 0.113, *P*=0.005). Importantly, GOLPH2 immunohistochemistry exhibited a lower level of intratumoral heterogeneity (25 *vs* 45%). Further, GOLPH2 upregulation was detected in 26 of 31 (84%) AMACR-negative prostate cancer cases. These data clearly suggest GOLPH2 as an additional ancillary positive marker for tissue-based diagnosis of prostate cancer.

The identification of sensitive and specific biomarkers in tissue and serum is of utmost importance to reduce the mortality of prostate cancer (Parekh *et al*, 2007). Expression arrays, SNP analyses and mass spectrometry are new tools for biomarker identification (Zheng *et al*, 2007). Such high-throughput analyses have recently identified new prostate cancer biomarkers, including, for example, HEPSIN, EZH2 and *α*-methyl-Co-racemase (AMACR) (Dhanasekaran *et al*, 2001; Jiang *et al*, 2001; Luo *et al*, 2001; Magee *et al*, 2001; Stamey *et al*, 2001; Varambally *et al*, 2002; Rhodes *et al*, 2003; Parekh *et al*, 2007). AMACR has first been found upregulated in prostate cancer by Xu *et al* (2000) using suppressive subtractive hybridisation, and AMACR antibodies have become available quickly thereafter (Jiang *et al*, 2001; Rubin *et al*, 2002). Thus far, it is the only new tissue biomarker of prostate cancer that has gained clinical acceptance. AMACR is frequently used in combination with the basal cell markers p63, CK5/6 and 34-*β*E12. In diagnostic histopathology, the absence of these basal cells, which usually line the periphery of normal prostate glands, is (with very rare exceptions) a defining criterion of invasive tumour growth (Brawer *et al*, 1985; Kaleem *et al*, 1998; Signoretti *et al*, 2000). However, it can be difficult to ascertain a cancer diagnosis in prostate needle biopsies. Use of an additional positive prostate cancer marker is desirable. AMACR immunohistochemistry can show dramatic pictures of strongly positive cancer glands infiltrating perfectly negative benign prostatic parenchyma and in these cases its use may turn a diagnosis of atypical glands into a straightforward diagnosis of cancer (Zhou *et al*, 2003, 2004; Epstein, 2004; Epstein and Herawi, 2006). However, it has been recognised that AMACR may be false-negative in up to 18% of prostate cancer foci on biopsies and even higher in some carcinoma subtypes (Epstein, 2004; Zhou *et al*, 2004).

Recently, *GOLPH2* mRNA expression has been reported to be upregulated in prostate cancer tissues (Luo *et al*, 2002; Lapointe *et al*, 2004; Kristiansen *et al*, 2005). GOLPH2 is a Golgi phosphoprotein of yet unknown function that has until very recently only been described in liver disease as a potential serum marker of hepatocellular carcinoma (Kladney *et al*, 2000, 2002a; Iftikhar *et al*, 2004; Marrero *et al*, 2005; Bachert *et al*, 2007). *GOLPH2* mRNA has recently been described as an integral part of a multiplex marker to detect prostate cancer from urine samples that even outperformed a prostate-specific antigen (PSA) blood test (Laxman *et al*, 2008).

In this study, we performed a comprehensive GOLPH2 protein expression analysis in a broad spectrum of normal and malignant tissues. Further, GOLPH2 expression patterns were studied in detail in different prostatic lesions. We demonstrate that GOLPH2 protein is upregulated in most prostate cancer cases. In addition to AMACR and p63, GOLPH2 antibodies will be helpful in the correct histological diagnosis of prostate cancer.

## MATERIALS AND METHODS

### Data mining of publicly available prostate cancer mRNA expression data

We interrogated the common gene expression databases, Oncomine and Arrayexpress, for differential expression of *GOLPH2* mRNA in human prostate cancer and normal tissue (Rhodes *et al*, 2004; Parkinson *et al*, 2007). We identified nine studies within Oncomine (Dhanasekaran *et al*, 2001, 2005; Luo *et al*, 2001, 2002; Vanaja *et al*, 2003; Lapointe *et al*, 2004; Varambally *et al*, 2005; Nanni *et al*, 2006; Tomlins *et al*, 2007) and one study within Arrayexpress (Liu *et al*, 2006). Altogether, these studies interrogated 305 samples of prostate cancer in combination with 148 of benign prostate tissues. From the Oncomine database, the normalised expression values for the nine studies were extracted and analysed using SPSS.

### Prostate cancer patients

Six hundred and fourteen prostate cancer patients who underwent radical prostatectomy between 1999 and 2005 were enclosed in this study. Patient age ranged between 43 and 74 years (median 62 years). Preoperative PSA levels ranged from 0.8 to 39 ng ml^−1^ (median 7.2). Forty-four patients (7.2%) had received gonadotropin-releasing hormone analogues at the discretion of the referring urologist before surgery (median 4 weeks, range 2–16 weeks). Clinical follow-up data were assessed annually. Prostate-specific antigen relapse-free survival time was available for 479 patients. The median follow-up time of all cases was 17 months (range 1–68 months). The median follow-up time of patients without a PSA relapse was 18 months (range 4–68 months). Forty-three patients (9%) experienced a PSA relapse after a median time of 5 months (range 1–52). The Gleason scores (GS) in the cohort were distributed as follows: GS 2–6: 217 (35.3%) GS 7: 291 (47.4%), GS 8–10: 106 (17.3%). Four hundred –and twenty cases had organ-confined carcinomas (pT2); 191 cases showed extracapsular tumour extension (pT3). The surgical margins were clear (R0) in 444 cases; 167 cases had positive margins (R1) and 3 cases were Rx. Use of this tissue has been approved by the Charite’ University Ethics Committee under the title ‘Retrospektive Untersuchung von Gewebeproben mittels immunhistochemischer Färbung und molekularbiologischer Methoden’ (‘Retrospective analysis of tissue samples by immunohistochemistry and molecular biological techniques’) (EA1/06/2004) on 20 September 2004.

### Screening tissue microarray construction

Formalin-fixed and paraffin-embedded material of a representative variety (185 spots) of normal and malignant human tissues and tumour cell lines was compiled and assembled on a single block, as described (Varga *et al*, 2006).

### Prostate tissue microarray construction

Formalin-fixed, paraffin-embedded tissue blocks of radical prostatectomy specimens were selected according to tissue availability for construction of a TMA. Each case was represented by five tissue cores. In all cases, benign prostatic hyperplasia (BPH) of the transitional zone, normal tissue from the peripheral zone, prostatic intraepithelial neoplasia (PIN), if present (otherwise another core from the peripheral zone), and two cores of invasive carcinoma, ideally of primary and secondary GS, were selected for TMA construction. The core diameter was 1.0 mm. All cases were arranged in 40 TMA recipient paraffin blocks.

### Immunohistochemistry

The TMA blocks were freshly cut (3 *μ*m) and mounted on superfrost slides (Menzel Gläser, Braunschweig, Germany). Immunohistochemistry was conducted with the Ventana Benchmark automated staining system (Ventana Medical Systems, Tucson, AZ, USA) using Ventana reagents for the entire procedure. To detect GOLPH2, two commercially available antibodies (mouse monoclonal, clone 5B10; Abnova Corp., Taipei, Taiwan, catalogue no. H00051280-M06, dilution 1 : 1000 and rabbit polyclonal; Abcam, Cambridge, UK, catalogue no. Ab22209, dilution 1 : 100) were diluted in a Ventana diluent. To detect racemase and p63, we created a cocktail of racemase (rabbit polyclonal; Biologo, Kronshagen, Germany, dilution 1 : 30) and p63 (clone mix 4A4/Y4A3; Neomarkers, Fremont, CA, USA, dilution 1 : 200) in a Ventana diluent. Primary antibodies were detected using the UltraVIEW DAB detection kit using the benchmarks CC1m- heat-induced epitope retrieval. For the racemase/p63 cocktail, the signal was further enhanced with the amplification kit. Slides were counterstained with haematoxylin, dehydrated and mounted.

### Evaluation of the immunohistochemical stainings

Chromogenic immunohistochemistry using both GOLPH2 antibodies was primarily conducted on a multitissue array constructed for antibody testing comprising 185 human tissue spots and cell lines. The immunostainings were evaluated by two genitourinary pathologists (GK, FFR) and one histopathology resident (CJ) simultaneously on a multiheaded microscope.

For both GOLPH2 and racemase, we evaluated staining intensity with a four-tiered system: 0 (negative), 1+ (weak), 2++ (moderate), 3+++ (strong) in benign tissue, PIN and invasive carcinoma. To also detect very subtle staining intensity differences, we further created a dichotomous (‘tumour>normal’) ratio to better indicate upregulation in tumour in comparison with adjacent normal tissue. Equal or less GOLPH2 staining intensity in carcinomatous tissue was reported as ratio 0, higher staining intensities than in normal glands were regarded as ratio 1.

Heterogeneity of marker expression in invasive carcinoma was also recorded and diagnosed if more than 25% of the tumour showed a variation of staining intensity exceeding one scoring category. P63 immunoreactivity of the racemase/p63 cocktail was sometimes used to clearly distinguish benign and malignant glands.

### Monoclonal and polyclonal GOLPH2 double staining by immunofluorescence

GOLPH2 is a Golgi protein. To better assess the specificity of the polyclonal and the monoclonal antibody, a double staining by immunofluorescence was conducted. Primary antibodies (mouse-anti GOLPH2, Abnova Corp., 1 : 4000; rabbit-anti GOLPH2, Abcam Ltd., 1 : 200) were coincubated on a de-paraffinised prostate tissue slide after heat-induced antigen retrieval (5 min, citrate buffer, pH 6.0, 110°C) at room temperature for 30 min. Binding was detected by fluorescence-labelled secondary antibodies (goat anti-rabbit-Alexa546 and goat anti-mouse-Alexa488, both from Molecular probes, catalog nos. A11010 and A11029) under a fluorescence microscope.

### Antibody preincubation with immunogenic peptide

To further assess antibody specificity, the monoclonal antibody was incubated with an excess of the immunogenic peptide provided by the antibody supplier (partial recombinant protein (NP_057632, 302 aa–402 aa) with GST, Abnova Corp.) at 4°C overnight before application to the control tissue (Figure 2F).

### Statistical analysis

Statistical analysis was performed using SPSS version 15.0. *P*-values <0.05 were considered significant.

## RESULTS

### GOLPH2 mRNA expression in prostate cancer

**Figure 1:**
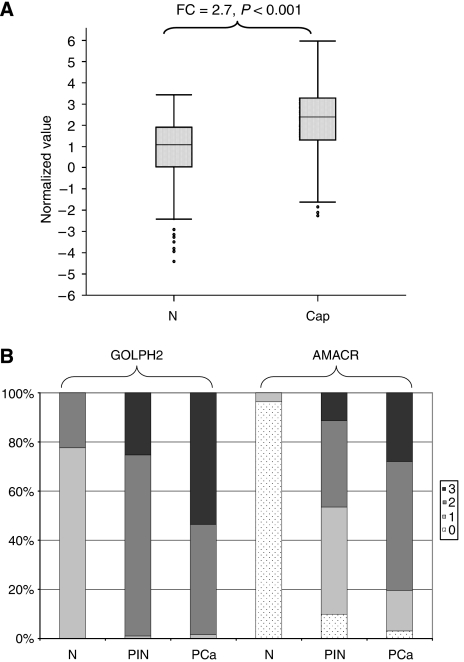
GOLPH2 expression in prostate tissues at mRNA and protein level. (**A**) Boxplot of the combined normalised expression values of the nine studies from Oncomine interrogating normal and cancerous prostate tissues. The fold changes and the respective *P*-values are indicated above the brackets. CaP=prostate cancer tissue; FC=fold change; N=normal prostate. The open circles indicate outliers. (**B**) Illustration of the progression of GOLPH2 (on the left) and AMACR expression (on the right) from normal tissue through PIN to invasive carcinoma (immunohistochemical data).

We have reported earlier that *GOLPH2* mRNA is overexpressed in microdissected prostate cancer epithelium compared with the adjacent normal prostate epithelium from the same patient by a fold change of 2.2 (Kristiansen *et al*, 2005). Liu *et al* (2006) described *GOLPH2* mRNA as overexpressed by a fold change of 3.14 in their samples (13 normal; 45 cancer), which did not correlate to tumour differentiation according to GS. A comprehensive analysis of the studies from Oncomine combining 260 samples from CaP and 135 from benign prostate normal revealed an overexpression of *GOLPH2* by a factor of 2.7 in prostate cancer (*P*<0.001, Figure 1A).

### GOLPH2 protein expression in normal and neoplastic human tissues

**Figure 2:**
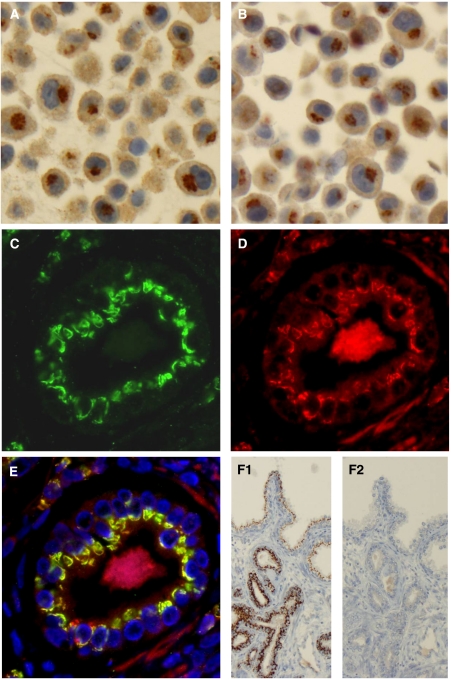
Characterisation of GOLPH2 antibodies. (**A** and **B**) Chromogenic immunocytochemistry of the paraffin-embedded melanoma cell line PF2000. Both antibodies (**A** – mouse monoclonal, Abnova; **B** – rabbit polyclonal, Abcam) show a strong semigranular perinuclear staining, which is suggestive of a Golgi pattern. (**C** and **D**) Immunofluorescent double staining of a prostate cancer gland using both GOLPH2 antibodies (**C** – mouse monoclonal, **D** – rabbit polyclonal). The signal of both antibodies is clearly located to the golgi apparatus, which can now be appreciated by the higher resolution of immunofluorescence. (**E**) The colocalisation of the immunoreactivity of both antibodies (plus DAPI staining), which shows that the polyclonal antibody (red signal) has a less favourable signal to background ratio. (**F1**) A GOLPH2 immunohistochemistry (monoclonal antibody) of prostate cancer tissue (lower part – malignant glands, upper part – normal glands) and (**F2**) a consecutive section of the same case was immunostained after preincubation of the antibody with an excess of the immunogenic GOLPH2 peptide, which abolishes immunoreactivity.

Both GOLPH2 antibodies showed identical stainings on a multitissue array comprising 185 tissue spots and cell lines (Figure 2A and B). Highest rates of GOLPH2 expression were seen not only in adenocarcinomas of the prostate, colon and breast, but also in renal cell cancer and hepatocellular carcinoma (Table 1). Prostate cancer showed the strongest staining.

**Figure 3:**
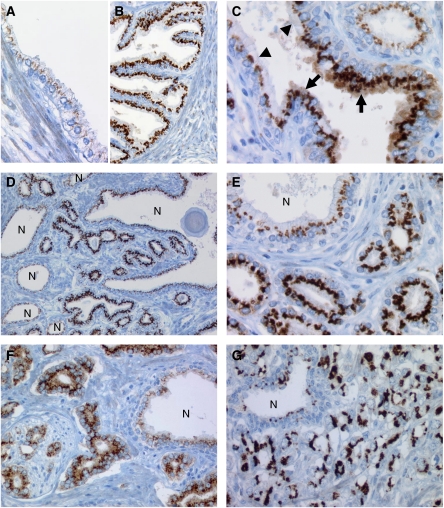
GOLPH2 expression in prostate tissues. (**A**) Normal secretory epithelium of normal prostate glands (immunoreactivity score 1+). (**B**) Hyperplastic gland with stronger GOLPH2 expression (score 2+). (**C**) Transition of normal epithelium (arrowheads) to high-grade PIN. Note prominent nucleoli (arrows). This PIN has a strong GOLPH2 immunoreactivity (3+) and shows an additional diffuse cytoplasmic staining. (**D**) Gleason 3+3=6 adenocarcinoma (central) infiltrating in between normal glands (marked ‘N’). Note the upregulation of GOLPH2 (3+) in comparison with normal glands. (**E**) Same case at a higher magnification. Note the characteristic Golgi pattern. (**F**) Gleason 3+3=6 adenocarcinoma, with a more diffuse cytoplasmic GOLPH2 staining (3+). Note neural invasion (lower left). (**G**) High-grade adenocarcinoma (Gleason score 3+4=7) with a strong and coarse GOLPH2 staining (3+).

**Figure 4:**
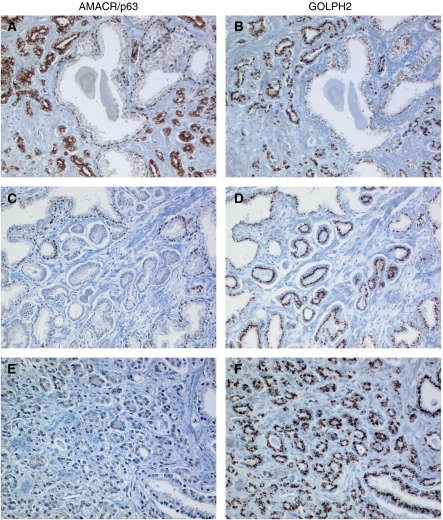
Comparison between AMACR/p63 and GOLPH2 immunohistochemistry. (**A**) AMACR expression in invasive cancer glands. Epithelium of normal glands, with a p63-positive basal cell layer, is AMACR-negative. (**B**) Sequential section showing GOLPH2 upregulation in matching cancer glands (score 2+); adjacent normal glands are weakly GOLPH2-positive (score 1+). (**C**) Shows an AMACR-negative example of invasive prostate cancer, whereas the same tumour has a significant upregulation of GOLPH2 (**D**) in comparison with normal glands (upper left corner, lower right corner). The case depicted in (**E**) and (**F**) has no included normal glands, but nonetheless a very strong GOLPH2 expression (3+) that is rarely seen in normal glands.

**Figure 5:**
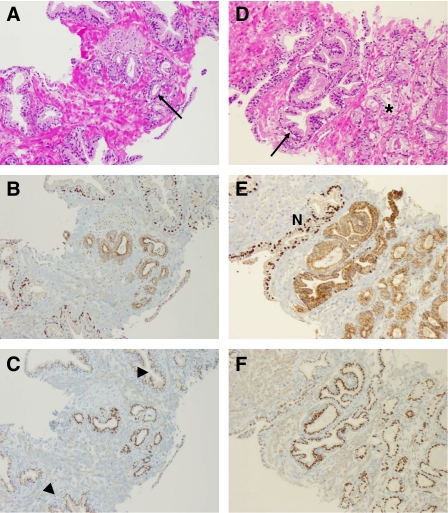
Two examples (**A**–**C**, **D**–**F**) of prostate needle biopsies (H&E, AMACR/p63, GOLPH2). (**A**) Prostate needle biopsy with a small focus of a Gleason 3+3 adenocarcinoma (arrow). Sequential sections of this focus show a lack of p63-positive basal cells and a moderate AMACR immunoreactivity (**B**). GOLPH2 is moderately strongly expressed in these glands, compared with adjacent normal glands (arrowheads), which have a weaker GOLPH2 staining (**C**). (**D**) Another example of a prostate needle biopsy with atypical glands, some are macroacinar (arrow), some (lower right) are smaller (^*^). (**E**) The AMACR/p63 cocktail demonstrates a continuous basal cell layer in larger normal gland on top (marked ‘N’), the macroacinar glands directly adjacent to it and the microacinar proliferates in the lower right corner have no basal cells. In between is a larger gland with a disrupted basal cell layer, probably diagnostic of a high-grade PIN. All these glands are strongly positive for AMACR and for GOLPH2 (**F**). It is of importance to note that in this case, both markers (AMACR and GOLPH2) do not differentiate between the high-grade PIN and the invasive carcinoma.

GOLPH2 expression can be observed in mesenchymal cells and epithelia, but with strongly differing intensities. As can be expected from a Golgi-associated protein, it shows a distinct semigranular dot-like staining pattern and is localised perinuclearly towards the cell apex in epithelia, whereas the rest of the cytoplasm is remarkably clear (Figure 2A and B, Figures 3, 4 and 5). To further cross-validate antibody specificity, a double immunofluorescent staining using both antibodies was conducted and demonstrated a clear colocalisation of the antigens in the Golgi apparatus (Figure 2C–E). Again, the monoclonal and the polyclonal antibody showed identical staining localisations. The monoclonal antibody was preferred for further immunostaining our prostate cancer cohort as it yielded slightly less background and a more intense signal at lower concentrations. In addition, the preincubation of the monoclonal antibody with an excess of recombinant GOLPH2 protein completely abolished immunoreactivity (Figure 2F).

### GOLPH2 immunostaining in prostate tissues

Perinuclear GOLPH2 expression is present in normal and neoplastic prostate glands with unequivocal upregulation in most hyperplastic and neoplastic glands in comparison with normal glands. Some cases of PIN and carcinoma, however, display an attenuation of the Golgi staining and an additional diffuse strong cytoplasmic immunoreactivity, which was seen in 68 cases (11.1%) (Figure 3C and F).

Normal prostatic glands show finely granular GOLPH2 staining, in some instances with an almost linear pattern (Figure 3A). The staining intensity is relatively weak yielding a more golden than brownish DAB precipitate. The median GOLPH2 intensity in normal tissue was 1+ (Figure 1B). Hyperplastic glands of BPH show a moderate-to-strong staining intensity (Figure 3B). Glands with slightly atypical epithelia also show a stronger immunoreactivity than the normal glands. This becomes even more pronounced in high-grade PIN (median intensity 2+) and invasive carcinoma (median intensity 3+) wherein the granules are much coarser and stain deeply brown, which yields a well-discernable contrast to adjacent benign glands (Figures 3C–G, 5C and F). The statistical differences between GOLPH2 expression in normal, PIN and carcinoma were highly significant (Figure 1B; Wilcoxon's signed rank test, *P*<0.001).

### GOLPH2 histopathology and survival

GOLPH2 protein expression in prostate cancer was not associated with pT stage, differentiation grade (GS) and preoperative PSA levels. There was no association with disease-free survival (Cox regression, relative risk 0.969, *P*=0.910).

### GOLPH2 as a potential tool for prostate cancer diagnosis

The most impressive finding of this expression analysis was a striking difference in GOLPH2 expression in normal and neoplastic prostate glands. In 237 cases (38.6%), GOLPH2 intensity was two scoring points higher in tumour epithelia than in normal glands; in another 324 cases (52.8%), tumoral GOLPH2 expression excelled by one scoring point; in 51 cases (8.3%), no differences between normal and tumour were noted; and in only two cases (0.3%), normal tissue showed a stronger GOLPH2 staining than adjacent tumour. In summary, 91.4% of cases showed an upregulation of GOLPH2 in the tumour by at least one scoring point. This rate is even higher in the separately scored ‘tumour>normal’ ratio. This is able to measure even subtle differences: 567 of 614 cases (92.3%) had a ratio of 1 and only 47 cases (7.7%) had a ratio of 0.

To characterise GOLPH2 as a new diagnostic tissue marker of prostate cancer, we conducted a careful comparison with the well-established AMACR immunohistochemistry. As expected, AMACR was found overexpressed in high-grade PIN (median score 1+) and invasive prostate cancer (median score 2+), whereas normal tissues were found to be negative (median score 0) (Figure 1B). AMACR overexpression in the tumour in direct comparison with adjacent normal tissue (‘tumour>normal’ ratio) was seen in 95% of cases. AMACR expression was significantly but not highly correlated to GOLPH2 expression (Table 2, Spearman rank correlation coefficient 0.113, *P*=0.005). However, both markers also showed remarkable differences, particularly, when the tumour/normal ratio of GOLPH2 and AMACR was considered. Here, 26 of 31 AMACR-negative cases (84%) were identified by GOLPH2. On the other hand, 42 of 47 cases (89%) without GOLPH2 upregulation were AMACR-positive. Five cases were concordantly negative and 541 cases were positive for both markers (Table 3). Four of the five cases, negative for both markers, were of higher GS. Examples of the comparison of AMACR and GOLPH2 expression in prostate tissues are shown in Figures 4 and 5.

The histologically evident intratumoral heterogeneity of prostate cancer is also reflected in biomarker expression. In this study, intratumoral AMACR and GOLPH2 heterogeneity of expression was also evaluated. AMACR has a considerably higher degree of heterogeneous expression (45% of cases) than GOLPH2 (25%). This heterogeneity (Figure 4E) can be troublesome in small tumour foci. In 43 cases, one of two TMA tumour cores was completely AMACR-negative, whereas the other core of the same case showed some immunoreactivity. Of these AMACR-negative cores, GOLPH2 was upregulated in 36 cases (84%).

The combination of GOLPH2 and AMACR showed expression of either marker in 99.2% of cancer cases, which advocates a combined use of AMACR and GOLPH2 as positive confirmative markers of prostate cancer.

## DISCUSSION

This is the first report on GOLPH2 (Golgi protein 73, GP73) protein expression in prostate tissues validated on a large cohort of clinically detected prostate cancer specimens following radical prostatectomy. We have recently shown that *GOLPH2* mRNA is among the top upregulated transcripts in prostate cancer (Kristiansen *et al*, 2005), which is in line with other profiling studies (Dhanasekaran *et al*, 2001, 2005; Luo *et al*, 2001, 2002; Vanaja *et al*, 2003; Lapointe *et al*, 2004; Varambally *et al*, 2005; Liu *et al*, 2006; Nanni *et al*, 2006; Tomlins *et al*, 2007). In our meta-analysis of publicly available expression data encompassing 260 prostate cancer cases, a mean fold change of 2.7 for *GOLPH2* upregulation in cancerous tissues was found. However, a detailed tissue-based *in situ* analysis of GOLPH2 protein in prostate tissues was lacking so far. Very recently, this widely acknowledged upregulation of *GOLPH2* was put into practise: Laxman *et al* (2008) included *GOLPH2* in a multiplex RT–PCR panel of markers composed of transcripts known to be overexpressed in prostate cancer, which, as a urine-based screening test, allows detecting prostate cancer with a higher sensitivity than a classical PSA blood test.

GOLPH2 is a 73-kDa Golgi apparatus-associated protein coded by the gene *GOLM1* located on chromosome 9q21.33 and was originally cloned from a library derived from liver tissue of a patient with adult giant-cell hepatitis (Kladney *et al*, 2000). The initial report also described GOLPH2 expression in a variety of other human tissues at RNA and protein level and demonstrated colocalisation of GOLPH2 with giantin, a type II Golgi membrane protein located at the *cis* and medial Golgi compartment. Structurally, GOLPH2 protein consists of a short cytoplasmic N terminus, a membrane-spanning region, some coiled-coil domains and a longer luminal C terminus with several potential glycosylation sites. The functions and the mechanisms of GOLPH2 regulation in normal and neoplastic tissues are still unclear. It can be generally assumed that it is either involved in post-translational protein modification, transport of secretory proteins, cell signalling regulation or simply maintenance of Golgi apparatus function. Functional assays are necessary to clarify whether GOLPH2 overexpression confers pro-tumorigenic properties to tumour cells and how it is regulated. First colocalisation experiments with GPP130, another Golgi marker, hinted at a differential colocalisation with GOLPH2 in normal and malignant prostate tissues, which deserves further study. GOLPH2 has several potential glycosylation sites and up to 75% of GOLPH2 secreted from hepatocytes is fucosylated, but so far the glycosylation patterns of GOLPH2 in malignant and normal prostatic epithelia have not been analysed (Norton *et al*, 2007).

In the liver cancer cell line HepG2, GOLPH2 was found strongly upregulated after adenoviral infection, which suggested GOLPH2 as a marker of viral infection in liver tissue and which was confirmed in following studies incorporating clinical samples (Kladney *et al*, 2002a, 2002b). More recently, GOLPH2 was found upregulated in the serum of patients with hepatocellular carcinoma (HCC) compared with healthy individuals and has been proposed as a new serum marker of HCC, which is more sensitive than *α*-fetoprotein (Block *et al*, 2005; Marrero *et al*, 2005). Apparently, GOLPH2-overexpressing hepatocytes secrete this normally membrane-bound Golgi protein after cleavage into the serum, which can be diagnostically utilised (Bachert *et al*, 2007). We can confirm the GOLPH2 expression in HCC; however, the finding that adenocarcinomas of the colorectum, the breast and the prostate showed equally strong or even stronger immunostainings argues against GOLPH2/GP73 as a HCC-specific tissue marker. This finding also implies that further serum analysis of non-HCC cancer patients, especially prostate cancer patients, is clearly necessary, before the role of GOLPH2/GP73 as a serum marker specific for HCC can be further established.

Histological diagnosis of prostate cancer mainly rests on the conventional parameters of morphological architecture and cytology. Prostate-specific antigen serum screening has led to an increase of prostate needle biopsies in the last two decades, which in turn increased the rate of difficult diagnostic situations (small carcinoma infiltrates *vs* benign mimickers of carcinoma) where immunohistochemical tests are necessary. Loss of basal cells is a hallmark of prostate cancer; hence, high molecular weight cytokeratins and p63 have become widely used basal cell tissue markers. However, even with a loss of basal cells, cancer diagnosis can be problematic in some cases. Additional markers of prostate cancer are desirable. So far only AMACR/racemase has gained wider acceptance as a positive marker of prostate cancer, although is has two well-known limitations: intratumoral heterogeneity, which was confirmed in 45% of our cases, and AMACR-negative carcinomas (Wang *et al*, 2006; Murphy *et al*, 2007). In our series, 31 completely AMACR-negative carcinomas (5%) and another 43 cases (7%), in which one of both tumour cores on the TMA was negative, were seen. In these 12% of cases, which might have been considered negative on a needle biopsy, an additional GOLPH2 immunostaining would have allowed a cancer diagnosis in 84% of cases. This is partially because of the considerably lower rate of intratumoral heterogeneity of GOLPH2, which was 25% in our series. These findings clearly advocate the use of GOLPH2 as an additional ancillary positive marker for the histological detection of prostate cancer. Comparable with the introduction of AMACR, we would expect that the number of unclear cases can be further lowered by GOLPH2, which would help to avoid costly and unnecessary rebiopsies (Jiang *et al*, 2004). Although GOLPH2 immunostaining is not as easy to read as an AMACR staining at first sight, mainly because of the physiological basal GOLPH2 expression in normal tissues, we think that the internal positive control of immunoreactivity in normal tissues can also be seen as an advantage. In addition, the characteristic Golgi pattern is another indicator of specific immunoreactivity, whereas a general overstaining of a slide is often more diffusely cytoplasmic.

In spite of our comprehensive description of GOLPH2 as a positive marker of malignancy, we would hesitate to recommend using GOLPH2 as the primary second-line antibody after basal cell markers for determining malignancy. First, its sensitivity is slightly lower (92.3%) than AMACR (95.0%), which is, of course, compensated for by its higher homogeneity. Secondly, and more importantly, definition of a positive test result requires adjacent normal glands for direct comparison. As high-grade PIN and hyperplastic benign glands can also show GOLPH2 upregulation, it can be difficult, or even impossible, to diagnose an atypical focus that lacks adjacent normal glands by GOLPH2 immunohistochemistry alone. The comparison with normal tissue is mandatory to obtain a valid result. Another caveat stems from the construction of our TMA, which has been compiled after central review of 640 fully embedded prostatectomy specimens to allow an immunohistochemical evaluation of representative normal and tumour tissue. Benign cancer mimickers, which can be particularly problematic to diagnose on needle biopsies, were not intentionally sampled. Further validation of the diagnostic value of GOLPH2 in rare cancer variants and benign cancer mimickers is necessary.

In summary, this study is the first to comprehensively confirm at protein level the GOLPH2 upregulation in prostate cancer, which has been suggested in preceding mRNA profiling studies. The high rate of GOLPH2 protein overexpression, which is also seen in AMACR-negative prostate cancer cases, suggests its use as an additional ancillary positive tissue marker of prostate cancer.

## Figures and Tables

**Table 1:** GOLPH2 expression in normal and neoplastic human tissues and cell lines

**Tissue/cell line (*n*)**	**GOLPH2−**	**GOLPH2+**	**GOLPH2++**	**GOLPH2+++**
Normal testis (2)	0	2	0	0
Seminoma (2)	0	1	1	0
Teratoma (2)	0	1	1	0
Placenta (2)	0	2	0	0
Invasive lobular breast carcinoma (4)	0	0	3	1
Invasive ductal breast cancer (4)	0	1	2	1
Cholangiocarcinoma (2)	0	0	1	1
Hepatocellular carcinoma (HCC) (2)	0	0	1	1
Lung adenocarcinoma (1)	0	0	1	0
Lung squamous cell carcinoma (1)	1	0	0	0
Lung small cell carcinoma (1)	0	1	0	0
Serous ovarian carcinoma (2)	1	1	0	0
Ovarian endometrioid carcinoma (1)	0	0	1	0
Ovarian mucinous carcinoma (1)	0	0	1	0
Endometrium endometrioid carcinoma (2)	0	1	0	1
Endometrium serous carcinoma (2)	0	1	1	0
Colon adenocarcinoma (4)	0	1	1	2
GIST (1)	0	0	1	0
Skin squamous cell carcinoma (2)	1	1	0	0
Merkel cell carcinoma (1)	0	1	0	0
Anaplastic oligodendroglioma (1)	0	1	0	0
Anaplastic astrocytoma (1)	0	1	0	0
Glioblastoma multiforme (1)	0	0	1	0
Thyroid papillary carcinoma (2)	0	1	0	1
Thyroid follicular carcinoma (1)	0	0	1	0
Thyoid anaplastic carcinoma (1)	0	0	0	1
Normal kidney (2)	0	2	0	0
Clear cell renal cell carcinoma (4)	0	0	0	2
Papillary renal cell carcinoma (2)	0	0	0	2
Urothelial carcinoma, bladder (4)	0	0	3	1
Adenocarcinoma, prostate (4)	0	0	1	3
Benign prostatic hyperplasia (2)	0	0	2	0
Normal liver (2)	0	2	0	0
Tonsils (3)	0	3	0	0
Non-Hodgkin's lymphoma (4)	0	2	2	0
Hodgkin's lymphoma (1)	1	0	0	0
Melanoma (1)	0	1	0	0
HA98 (2) (melanoma)	0	0	2	0
HN2004 (2) (melanoma)	0	0	0	2
PF2000 (2) (melanoma)	0	0	2	0
MET5A (2) (mesothelioma)	0	0	2	0
SW480 (2) (colon cancer)	0	2	0	0
786-O (2) (renal cell cancer)	0	0	0	2
H69 (2) (lung cancer)	0	0	2	0
MCF-7 (2) (breast cancer)	0	0	2	0
SK BR 7 (2) (breast cancer)	0	2	0	0
HELA (2) (cervical cancer)	0	2	0	0
PC3 (2) (prostate cancer)	0	2	0	0
293-T (2) (human embryonal kidney)	0	2	0	0

**Table 2:** GOLPH2 protein expression in prostate cancer

	**GOLPH2 expression**			
	**1+**	**2+**	**3+**	***P*-value**
All cases	10 (1.6%)	275 (44.8%)	329 (53.6%)	
				
*Age*				0.321
⩽62	6 (1.9%)	143 (46.3%)	160 (51.8%)	
>62	4 (1.3%)	132 (43.3%)	169 (55.4%)	
				
*Pre-OP PSA* a				0.475
⩽10 ng ml^−1^	5 (1.1%)	197 (44.6%)	240 (54.3%)	
>10 ng ml^−1^	5 (3.0%)	73 (44.2%)	87 (52.7%)	
				
*pT status*				0.267
pT2	8 (1.9%)	194 (45.9%)	221 (52.2%)	
pT3/4^b^	2 (1.0%)	81 (42.4%)	108 (56.5%)	
				
*Gleason score*				0.264
3–6	1 (0.5%)	92 (42.4%)	124 (57.1%)	
7	7 (2.4%)	136 (46.7%)	148 (50.9%)	
8–10	2 (1.9%)	47 (44.3%)	57 (53.8%)	
				
*Residual tumour* c				0.457
R0	6 (1.4%)	206 (46.4%)	232 (52.3%)	
R1	4 (2.4%)	68 (40.7%)	95 (56.9%)	
				
*AMACR expression*				0.005
0	2 (10.5%)	7 (36.8%)	10 (52.6%)	
1+	3 (3%)	53 (52.5%)	45 (44.6%)	
2+	2 (0.6%)	152 (47.2%)	168 (52.2%)	
3+	3 (1.7%	63 (36.6%)	106 (61.6%)	

Abbreviations: AMACR=*α*-methylacyl-CoA racemase; Pre-OP PSA=preoperative PSA; pT-status=tumour stage.

a Preoperative PSA was not available for seven cases.

b One case was pT4.

c Three cases were Rx.

**Table 3:** Tumour/normal ratio of AMACR and GOLPH2 expression in prostate cancer

	**GOLPH2 tumour>normal**		
	***No***	***Yes***	**Total AMACR**
**AMACR tumour>normal**			
**No**	5	26	31 (5%)
**Yes**	42	541	583 (95%)
**Total GOLPH2**	47 (7.7%)	567 (92.3%)	

Abbreviation: AMACR=α-methylacyl-CoA racemase.
